# Meninigiomas of the Craniocervical Junction – A Distinctive Subgroup of Meningiomas

**DOI:** 10.1371/journal.pone.0153405

**Published:** 2016-04-12

**Authors:** Lasse Dührsen, Pedram Emami, Jakob Matschke, Tammam Abboud, Manfred Westphal, Jan Regelsberger

**Affiliations:** 1 Department of Neurosurgery, Universitätsklinikum Hamburg-Eppendorf, Hamburg, Germany; 2 Institute of Neuropathology, Universitätklinikum Hamburg-Eppendorf, Hamburg, Germany; Heinrich-Heine University, GERMANY

## Abstract

**Objective:**

Meningioma of the cranio-cervical junction is a rare diagnosis and demand a thorough surgical planning as radical excision of these tumors is difficult. In this context recurrence is most likely due to regrowth of residual tumor. The aim of this study was to evaluate the clinical course of patients operated for craniocervical meningioma (CCM) and to investigate the histological features of these tumors and their impact on recurrence rate.

**Methods:**

All patients who were operated for CCM at our institution between 2003 and 2012 were identified. Presenting symptoms, MRI findings, surgical approaches and recurrence rate were reviewed retrospectively using medical charts. Histological features of the included tumors were studied focusing on subtypes and MIB-1 immunoreactivity and compared with MIB-1 immunoreactivity in an age and gender-matched control group of patients with supratentorial meningioma.

**Results:**

18 patients with CCM with a mean age of 56.2 years and median follow-up of 60 months were included in the study. Sensory or motor deficit was the most frequent presenting symptom followed by neck pain and lower cranial nerve palsy. Simpson grade II resection was achieved in 16 patients and Simpson grade III resection in two patients. Mortality, morbidity and recurrence rates were 16.7%, 5.5% and 5.5%, respectively. According to the WHO-grading all were found to be grade I meningiomas. Histological subtypes included meningotheliomatous (10), transitional (2), fibrillar (2), angiomatous (3) and secretory (1) meningioma. The mean MIB-1 labeling index in the study group was significantly higher than in the control group, (7.2% and 3.6%, respectively), p < 0.05. There was no correlation between MIB-1 levels and tumor recurrence.

**Conclusions:**

CCM seems to have a benign character. Despite a significantly higher MIB-1 index, a high rate of recurrence was not observed. Therefore, approaches with high morbidity are not justified. Nevertheless, in view of the challenging approaches with limited access to the lesion, CCM should be considered a distinctive clinical subgroup.

## Introduction

Meningiomas of the craniocervial junction present a unique and rare diagnosis among meningiomas[1]. They originate from the meninges of the lower part of the clivus and the upper edge of the axis, laterally from the jugular tubercle to the upper aspect of the C-2 lamina. Approximately 70% of all tumors in the craniocervical junction are meningiomas of benign origin[2]. Complete resection should be the primary goal of surgery but is often difficult to achieve due to their close relationship to critical vascular structures, the brainstem and cranial nerves. Therefore, Simpson grade I resection is rarely achieved, which is why a higher risk of recurrence might be expected [3, 4].

The aim of our work was to evaluate the clinical course of patients operated for craniocervical meningioma (CCM) and to investigate the histological features of these tumors and their impact on recurrence rate.

## Methods

All patients treated between 2003 and 2012 with the diagnosis of CCM were included in our study. Medical charts and records were reviewed retrospectively to determine their clinical symptoms, neuroradiological assessment, surgical approaches and recurrence rate.

### Histological analysis

For immunohistochemistry, 4 μm slides of formalin-fixed and paraffin-embedded tumor tissue were stained on an automated Ventana HX system (Ventana-Roche Medical systems, Tucson, AZ, USA) following the manufacturer´s instructions. To determine the proliferative activity, the MIB1-antibody was used (Neo-Markers, RM 9106-S; Dilution 1:1000). Positively stained tumor cell nuclei were counted in 3 adjacent high-power fields measuring 0.19 square millimeter each and the percentage to all tumor nuclei was calculated (Ki67-labeling index). Histological specimens were evaluated with special attention to atypical features like increased cellularity, sheeting, necrosis, prominent nucleoli and nuclear-cytoplasmic ratio.

An age and gender-matched control group of patients who were operated for supratentorial meningioma, WHO grade I, was identified from our histological database and the MIB-1 immunoreactivity was determined for this group.

GraphPad Prism version 5.0 (GraphPad Software, La Jolla, Calif., USA) was used to analyse the data.

### Ethic statement

The study was approved by the local ethic committee at the medical council of the state of Hamburg (Ethik-Kommission der Ärztekammer Hamburg—WF-065/13). Patient information was anonymized and de-identified prior to analysis.

## Results

18 patients, 4 male and 14 female, with a mean age of 56.2 years (range 27–79 years) at presentation were included in the study. The median length of follow-up was 60 months (range 3–72 months). Initial symptoms leading to admission were pain (n = 3), sensory or/and motoric disability (n = 11) accompanied by lower cranial nerve palsies in three patients. An incidental finding was seen in one patient.

**Fig 1:**
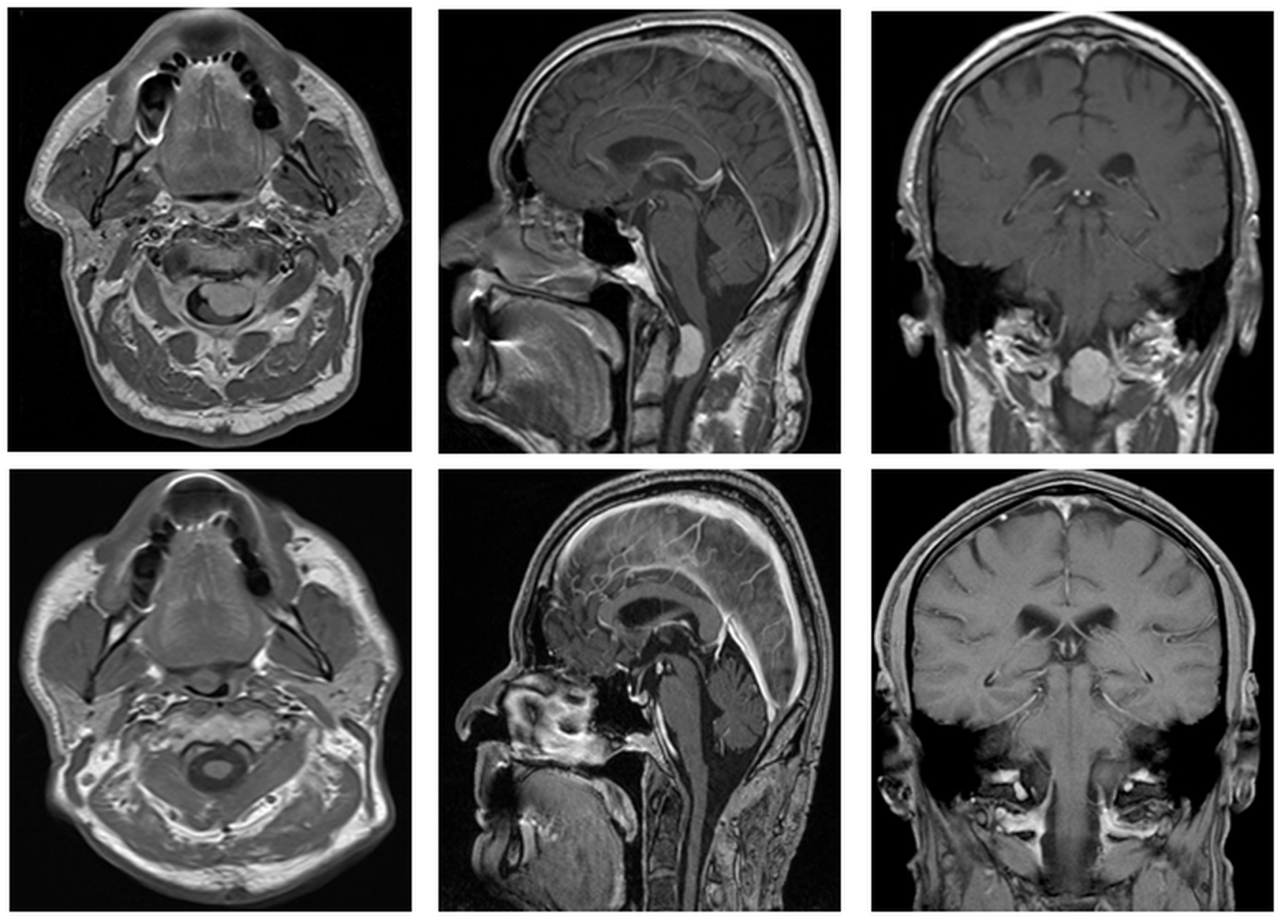
Pre- and postoperative MRI of craniocervical-menigioma which is operated on by a suboccipital midline approach with left lateral extension. Axial and sagittal MRI showing distortion and compression of the brain stem. With increasing growth of these lesions a surgical ‘door’ is opened thus allowing to attack the tumor in a less invasive manner. In our experience far lateral approaches with resection of the condyle are rarely, if at all, necessary in meningiomas due to their slow progression accompanied by minor clinical symptoms. But positioning of the patient may be already of great danger as inclination of the head will most likely increase the bending force on the brain stem, especially in large tumors. Therefore intraoperative SSEP and MEP monitoring are mandatory in this surgical area and should run while the patient is brought into the prone position thus minimizing the overall morbidity of this procedure.

Tumors were located between C0 and C2 in ten patients, at the level of foramen magnum in three patients and at the level of clivus, extending caudally in five patients. Of the upper cervical tumors, nine were located ventrally to the medulla and two lateral to it. The surgical approaches included retrosigmoidal (n = 1), suboccipital midline (n = 11), suboccipital midline with lateral extension (n = 6), with additional laminectomy of C1 (Fig 1). Intraoperative neuromonitoring using motor and sensory evoked potentials was performed during all procedures and showed no significant alteration.

In 16 patients complete removal of the tumor with coagulation of dural attachment (Simpson grade II) could be achieved via the suboccipital midline approach. Complete removal with partial coagulation of the dural attachment was achieved in the other two patients (Simpson grade III).

### Histological analysis

**Fig 2:**
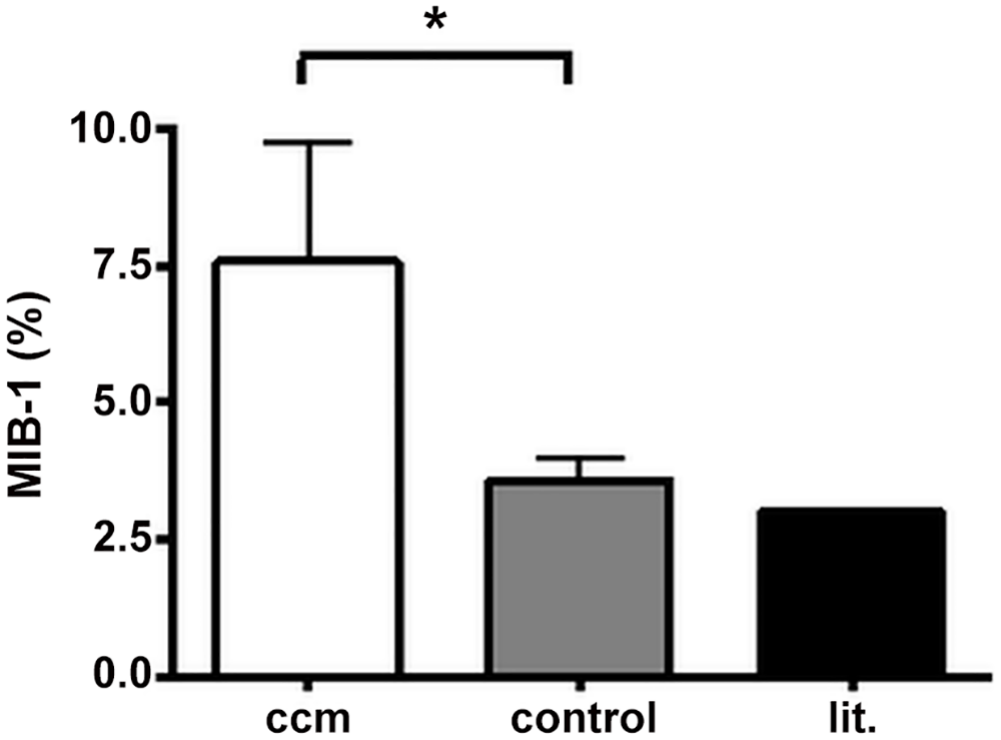
MIB-1 index is highly significant in CCM compared to age and gender controls (* = p < 0.05; student’s t-test; ccm: cranio-cervical meningioma; lit: literature).

According to WHO classification all tumors were grade I. None of the tumors showed atypical features. Histological subtypes were found to be meningotheliomatous (n = 10), transitional (n = 2), fibrillar (n = 2), angiomatous (n = 3) and secretory (n = 1) meningiomas. MIB-1 index showed a mean value of 7.2% (range from 2–31%, Fig 2) and was significantly higher than MIB-1 index in the control group with a mean of 3.6% (range from 1–7%; p<0.05).

### Postoperative course and follow-up

The morbidity rate was 16.7%. During postoperative stay on the ICU, one patient developed a subdural hematoma probably due to a postoperative newly diagnosed pancytopenia. The hematoma had to be evacuated and the patient could be discharged after a prolonged hospital stay without new deficit. One patient developed meningitis and was treated successfully with antibiotics. One patient complained of dysphagia. This subsided spontaneously and the patient recovered completely within a few weeks. One patient died due to postoperative pneumonia induced by prolonged mechanical ventilation leading to a mortality rate of 5.5%. At follow-up, one patient had two recurrences (Simpson grade II, MIB-index 5%) after one and three years.

## Discussion

Our cohort shows a clear preference for females, with a ratio 3.5 to 1, which is congruent with the literature [5, 6]. The presenting symptoms were unspecific but common for tumors in this location [7]. The tumor origin and elongation were not unusual compared to others [8]. One patient experienced recurrence of the tumor after one and three years after initial treatment. The tumor was initially resected Simpson grade II which in itself is a risk factor for tumor recurrence. In regular MRI controls there was no progression in a 7 years follow-up period. Postoperative complications were subdural hematoma, meningitis and dysphagia with an overall rate of transient morbidity of 16.5%. Compared to the literature this is rather modest with reported rates up to 100% [9]. In comparison to other approaches, namely far-lateral and posterolateral suboccipital retrocondylar approach, the rate of morbidity in our series is low and results for grade of resection are comparable [3, 10]. If the tumor is eligible in size we even prefer to approach it via a tubular system [11].

On histological examination special attention was paid to atypical features since the occurrence of single features, even when the tumor overall is diagnosed WHO grade I, seem to influence the risk of recurrence [12]. In none of the tumors in our study atypical features were found. Consequently, we looked at the proliferation marker Ki-67, MIB-1, quantifying their proliferative potential, beside the histological grade [13]. The Ki-67/MIB-1 monoclonal antibody is commonly used. It is reactive against the nuclear antigen Ki-67 expressed during cell cycle (G 1, S, G 2 and M) but absent in G 0 [14]. Several studies were carried out to investigate how MIB-1 labelling indices could help to predict recurrences [15]. Even though there is no standardized test or an international agreement where to set the threshold for an elevated MIB-1 labeling index, it is generally accepted that grade I meningiomas have a MIB-1 index of around 3% [16, 17]. In our study the MIB-1 index in meningiomas of the craniocervical junction was significantly higher (mean 7.6%) than in an age and gender matched controlgroup of supratentorial meningiomas WHO grade I (MIB-1 index mean 3.6%, Fig 1). This would explain a high recurrence rate of tumors originating from the craniocervical junction as reported by others [18]. Even though mean MIB-1 index in our study population is much higher than in controls described in the literature for grade I meningiomas we did not experience a high recurrence rate for our patients. One explanation could be a rather short period of follow up for this tumor entity. Futhermore, there is evidence that Simpson grading and the finding of single atypical features in histological specimen are responsible for recurrence in otherwise benign meningiomas [12]. Two of our meningiomas had MIB-1 indices as high as 29 and 31%, respectively, but showed no extraordinary clinical course. Moller et al. also did not find a significant relation between MIB-1 index and recurrence rate of meningiomas [19]. Two large retrospective series supported these findings and showed no obvious correlation between MIB-1 index and the risk for tumor recurrence [17, 20]. This is congruent with our results where tumor recurrence was seen in just one patient after one and three years. Both recurrences were operated on and have not shown any relapse since (Simpson grade II, MIB-1 5%). Even though the tumors showed criteria for recurrence like Simpson grade II and III and an elevated MIB-1 index they did not behave in an aggressive manner. This benign behavior was observed in other skull base meningiomas as well [21, 22]. The prognostic value of MIB-1 index for tumor recurrence is apparently only related to a certain subgroup of mengiomas. A more technical but often quoted explanation could be that the counting technique and cut off levels are not defined accurately which leads to differences between laboratories[23]. 3% cut off level may be reasonable at present but needs to be validated as discrepancies lead to different interpretation.

## Conclusions

CCM remain a challenging field for neurosurgery. A cure can be achieved surgically in these benign lesions, however limited access may complicate this attempt. Due to their exceptionally benign behavior complete resection should not be forced. The suboccipital midline approach is associated with a low rate of morbidity and therefore should be used to avoid surgery related complications. The MIB-1 index seems to be of no prognostic value in CCM regarding the risk of recurrence. This seems to suggest that CCM are a special clinical entity where a precise histopathological work-up for larger series is needed.
